# Monitoring C–C coupling in catalytic reactions via machine-learned infrared spectroscopy

**DOI:** 10.1093/nsr/nwae389

**Published:** 2024-11-04

**Authors:** Li Yang, Zhicheng Zhao, Tongtong Yang, Donglai Zhou, Xiaoyu Yue, Xiyu Li, Yan Huang, Xijun Wang, Ruyun Zheng, Thomas Heine, Changyin Sun, Jun Jiang, Sheng Ye

**Affiliations:** Institutes of Physical Science and Information Technology, School of Chemistry & Chemical Engineering; Engineering Research Center of Autonomous Unmanned System Technology (Ministry of Education), Anhui Provincial Engineering Research Center for Unmanned System and Intelligent Technology, School of Artificial Intelligence, Anhui University, Hefei 230601, China; Helmholtz-Zentrum Dresden-Rossendorf, Dresden 01328, Germany; Theoretical Chemistry, Technische Universität Dresden, Dresden 01069, Germany; Institutes of Physical Science and Information Technology, School of Chemistry & Chemical Engineering; Engineering Research Center of Autonomous Unmanned System Technology (Ministry of Education), Anhui Provincial Engineering Research Center for Unmanned System and Intelligent Technology, School of Artificial Intelligence, Anhui University, Hefei 230601, China; Key Laboratory of Precision and Intelligent Chemistry, School of Chemistry and Materials Science, University of Science and Technology of China, Hefei 230088, China; Key Laboratory of Precision and Intelligent Chemistry, School of Chemistry and Materials Science, University of Science and Technology of China, Hefei 230088, China; Key Laboratory of Precision and Intelligent Chemistry, School of Chemistry and Materials Science, University of Science and Technology of China, Hefei 230088, China; Key Laboratory of Precision and Intelligent Chemistry, School of Chemistry and Materials Science, University of Science and Technology of China, Hefei 230088, China; Key Laboratory of Precision and Intelligent Chemistry, School of Chemistry and Materials Science, University of Science and Technology of China, Hefei 230088, China; Key Laboratory of Precision and Intelligent Chemistry, School of Chemistry and Materials Science, University of Science and Technology of China, Hefei 230088, China; Institutes of Physical Science and Information Technology, School of Chemistry & Chemical Engineering; Engineering Research Center of Autonomous Unmanned System Technology (Ministry of Education), Anhui Provincial Engineering Research Center for Unmanned System and Intelligent Technology, School of Artificial Intelligence, Anhui University, Hefei 230601, China; Helmholtz-Zentrum Dresden-Rossendorf, Dresden 01328, Germany; Theoretical Chemistry, Technische Universität Dresden, Dresden 01069, Germany; Institutes of Physical Science and Information Technology, School of Chemistry & Chemical Engineering; Engineering Research Center of Autonomous Unmanned System Technology (Ministry of Education), Anhui Provincial Engineering Research Center for Unmanned System and Intelligent Technology, School of Artificial Intelligence, Anhui University, Hefei 230601, China; Key Laboratory of Precision and Intelligent Chemistry, School of Chemistry and Materials Science, University of Science and Technology of China, Hefei 230088, China; Institutes of Physical Science and Information Technology, School of Chemistry & Chemical Engineering; Engineering Research Center of Autonomous Unmanned System Technology (Ministry of Education), Anhui Provincial Engineering Research Center for Unmanned System and Intelligent Technology, School of Artificial Intelligence, Anhui University, Hefei 230601, China

**Keywords:** chemical evolution, machine learning, spectroscopic descriptor, dynamic monitoring, catalytic reaction

## Abstract

Tracking atomic structural evolution along chemical transformation pathways is essential for optimizing chemical transitions and enhancing control. However, molecule-level knowledge of structural rearrangements during chemical processes remains a great challenge. Here, we couple infrared spectroscopy as a non-invasive method to probe molecular transformations, with a machine-learned protocol to immediately map the spectroscopic fingerprints to atomistic structures. From the theoretical perspective, we demonstrate it here with the example of C–C coupling in catalytic reactions, elucidating various structural conformations along dynamic trajectories. Within the transferable application to the specific CO–CO dimerization reaction, the structural and energetic variations of the critical chemical species could be identified via infrared spectroscopy. This approach extends the power of spectroscopy from fingerprinting chemical configurations to using them for assigning dynamic structural information.

## Teaser

The authors propose a novel machine-learned protocol to accurately monitor the atomistic structural transformations in chemical evolution via infrared spectroscopy descriptor.

## INTRODUCTION

Gaining insights into dynamic structural rearrangements is crucial for unraveling the complexities of chemical evolution and manipulating reactions with precision [1–3]. This pursuit holds immense significance across various scientific fields, including catalysis, materials science, and physical chemistry [4,5]. However, the inherent overwhelming complexity of chemical transformations often thwarts the facile detection and identification [5,6]. Even in cases of seemingly simple processes such as carbon–carbon (C–C) coupling, molecules pass through diverse configurations, presenting a formidable challenge in precisely tracking atomistic structural mobility and energetic variations [7]. Such limited information recognition hampers a comprehensive understanding of the dynamic processes of chemical transformations for further developments. Therefore, a simple yet efficient strategy to obtain an atomic-scale image of chemical evolution is highly desirable.

Being intrinsic ‘fingerprints’, spectroscopic tools are commonly used to probe the adsorbate-surface interactions, potentially paving strategies to identify the dynamic reaction processes [8,9]. Among these, non-invasive infrared (IR) spectroscopy enables the monitoring of vibrational excitations that are sensitive to specific structural configurations, providing detailed insights into the active sites and reaction pathways [5,10]. Nevertheless, correlating infrared spectral signals with high-dimensional atomistic structural information is still complex and demanding in both experimental and theoretical research. For the dynamic evolution procedures or chemical reactions, the continuous variations of configurations make it even more difficult to relate spectral signals and untangle the corresponding chemical structure. This limits the applicability and versatility of the spectroscopic techniques in the precise identification of chemical processes.

Recently, machine learning (ML) has emerged as a powerful tool for analyzing and interpreting the surface structural features and their chemical attributes, offering a promising avenue to swiftly pinpoint the intricate evolution processes [11]. Through sufficient data-driven training, ML can efficiently uncover the nature of active sites and unravel reaction pathways [12]. To ensure reliable predictions with ML models, it is crucial to use appropriate descriptors that are closely correlated with the target properties, highly extensive, and readily obtainable [13–15]. Therein, IR spectroscopy emerges as an advantageous descriptor for structural discernment owing to its availability both experimentally and theoretically and high sensitivity to surface structure information [13,14,16]. Thus, monitoring the structural evolution of dynamic processes involving chemical reactions should be possible via an IR spectroscopy-informed ML approach.

**Figure 1.**
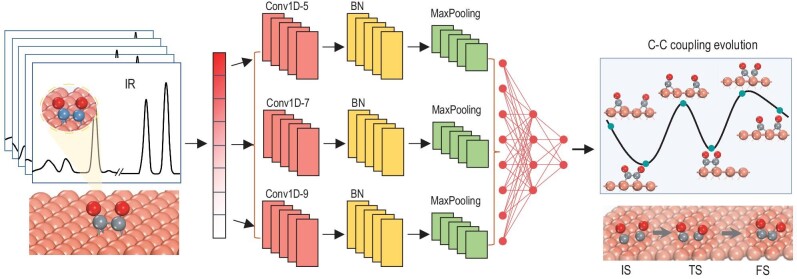
Schematic illustration of the machine learning protocol for monitoring the C–C coupling in catalytic reactions via IR spectroscopy. Brown, gray and red balls stand for Cu, C and O atoms, respectively. Conv1D-5/7/9 represent one-dimensional convolution with various sizes, BN stands for batch normalization, IS, TS and FS are the initial, transition and final states along the reaction pathway.

In the present work, we propose an ML-based framework to efficiently and accurately monitor the chemical evolution processes using IR spectroscopy. To intuitively illustrate this approach, C–C coupling involving the interaction of two adjacent CO intermediates in catalytic reactions was selected as a representative demonstration (Fig. 1). This process is crucial for converting excess CO_2_ into high-value chemicals, yet molecular insights into this dynamic evolution procedure remain poorly understood [17]. Furthermore, from a modeling perspective, this process can readily stand out to be a typical paradigm due to the well-defined CO infrared peaks, which often occur in a spectral region free of interfering signals [18,19]. Effective C–C coupling demands sufficient interaction between CO intermediates, typically characterized by a C–C distance ∼1.50 Å [7,20,21]. Convolutional neural network (CNN) was employed to recognize and extract the spectroscopic features, enabling the delineation of atomistic structures and chemical interactions in catalytic systems. We will show here that this network can accurately predict local atomistic structures of the active surface evolution and the energetic variations by deciphering IR spectroscopy. In this way, the structural rearrangements during C–C coupling are recapitulated in detail. By applying the trained ML model specifically to the CO–CO dimerization reaction, the crucial configurations and corresponding energy barriers are available. The predicted promotion tendency for CO–CO dimerization via metal dopant agrees well with previous literature, confirming the reliability of the ML model. Additionally, the distribution ratio of the associated configurations can be discerned within the spectroscopic signals, offering the potential to recognize the configuration assignment during the reaction. Altogether, our approach fuses IR spectroscopy, first-principles calculations, and ML to provide a simple yet effective venue to track structural evolution in the C–C coupling process.

## RESULTS AND DISCUSSION

### Data generation and analysis

**Figure 2.**
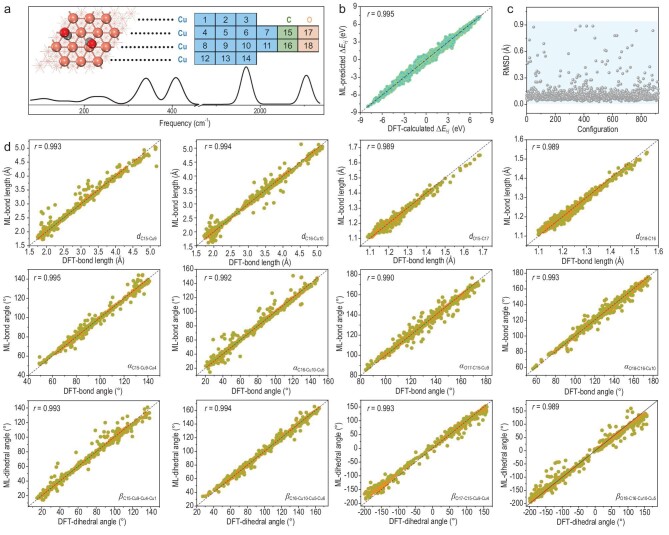
ML prediction of structural and energy information. (a) Scheme of the structure consisting of two adsorbed CO molecules and the surrounding Cu active sites for IR spectroscopy calculation. Critically investigated atoms are labeled for supervised learning. (b) Comparison of ML-predicted and DFT-simulated energy differences (ΔE*_i-j_, i* and *j* stand for two random structures of the extracted configurations) during C–C coupling evolution. (c) RMSD between ML-predicted and DFT-simulated structures of two adsorbed CO molecules and the surrounding active sites for the examined configurations. (d) ML prediction performance of the most relevant structural information (bond lengths (upper), bond angles (middle) and dihedral angles (lower)) for the two adsorbed CO molecules.

Owing to the intrinsic moderate adsorption properties of reaction intermediates, metal Cu has long been regarded as the benchmark and star material for CO_2_ conversion [22]. Therefore, in our study, C–C coupling on the typical and extensively-researched Cu surface was investigated as the representative paradigm (Fig. 2a). *Ab initio* molecular dynamics (AIMD) calculations (see Methods) were conducted to simulate the dynamic evolution process, where temperature and solvent water layers were set to closely match the experimental conditions [23,24]. A total number of 45 000 snapshots were generated over more than 22.5 ps of dynamic evolution, and each snapshot represents a specific configuration involving two adsorbed CO molecules (either separated or bound). To evaluate the structural variety of these geometries, the root mean square deviation (RMSD) was calculated with the first configuration as reference. As shown in Fig. S1a, some RMSD values extend to more than 3.5 Å, indicating significant structural mobility during the dynamic evolution. Such structural diversity was further confirmed by the considerable energy deviations, ensuring an ample sampling of various configurations during C–C coupling (Fig. S1b). From these AIMD snapshots, we extracted 9 500 configurations at 20 fs intervals as the investigation domain to mitigate the potential occurrence of correlated structures. Calculations of vibrational frequencies and density analysis were performed on these extracted configurations, wherein 9 369 datasets were obtained after filtering out some non-converged systems for the subsequent ML training.

### Machine learning architecture design

A CNN consisting of multiple blocks and a fully connected layer, implemented in the PyTorch deep learning framework [25], was designed to predict the target structural and energetic information with the calculated IR spectroscopy (involving vibrational frequencies and density as input, see Supplementary data for details). The investigated structure of two adsorbed CO molecules and the surrounding active sites were divided into 48 internal coordinates including bond lengths, bond angles and dihedral angles as the ML output (Fig. 2). Each CNN block contains three branches, with a convolutional layer, a ReLU activation function, a max pooling layer, and a batch normalization layer. The convolutional filters in these three branches have sizes of 5, 7 and 9 [26]. The outputs from three branches of each CNN block are concatenated along the channel dimension, and a 1 × 1 convolutional layer is applied to reduce the number of output channels. A squeeze-and-excitation (SE) block is employed, consisting of a global average pooling layer followed by two fully connected layers and a sigmoid activation function [27]. This SE block selectively weights the importance of each feature map. Our ML architecture utilizes multi-branch CNNs to capture diverse local features with squeeze-and-excitation layers for channel-wise attention, which learn discriminative spectral features effectively and adaptively [28].

### ML prediction of structural and energetic information

The predictive accuracy of the ML model is evaluated using the Pearson coefficient (*r*), and its robustness is validated through standard cross-validation procedures. As shown in Fig. 2b, by deciphering the IR spectral data, the ML-predicted energy differences for two random structures show excellent agreement with the DFT-calculated values, indicating the model's effectiveness in tracking energy variations during CO–CO coupling. Additionally, the essential structural details including bond lengths, bond angles and dihedral angles for the two adsorbed CO molecules and their surrounding active sites are predicted with high accuracy (*r* > 0.971). The average RMSD between the ML-predicted and DFT-simulated structures is around 0.13 Å, indicating that the ML model can successfully reproduce the atomistic positions of critical local structures during the C–C coupling process (Fig. 2c, d and Figs S2–S4).

### Monitoring C–C coupling evolution process

To further demonstrate the robustness and applicability of our approach, we performed an additional, independent AIMD simulation of C–C coupling with a different initial configuration as the validation set for ML model. Taking the initial configuration as reference, the RMSD for the CO molecules extends to more than 1.5 Å, indicating significant structural diversity (Fig. S5a). Moreover, the distance between two carbon atoms varies widely, suggesting continuous CO–CO dimerization and separation during the dynamic evolution (Fig. S5b).

**Figure 3.**
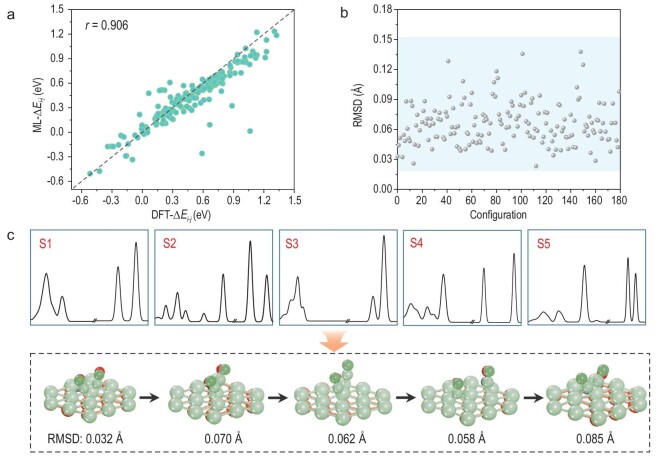
Monitoring C–C coupling evolution. (a) ML prediction performance for energy differences between two random structures for the extracted 180 configurations. (b) RMSD between ML-predicted and DFT-simulated structures for two adsorbed CO molecules and the surrounding active sites. (c) Monitoring C–C coupling evolution by deciphering IR spectra. Five representative states have been selected for demonstration. The solid structural models are the ML-reproduced atomic configurations, while the green transparent ones stand for DFT-simulated systems.

The energy and geometric structure of the extracted 180 configurations at equal intervals were then predicted with the trained ML model. As illustrated in Fig. 3a, the ML-predicted energy differences closely align with the DFT-calculated ones with a Pearson coefficient of 0.906. Only few outliers have been observed, indicating the robustness of the ML energy predictions. The low RMSD values (averaging at 0.07 Å) between ML-reproduced and DFT reference configurations indicate pronounced structural uniformity and underscore the feasibility of probing the high-dimensional geometric information using IR spectroscopy (Fig. 3b). For elaborate illustration, five successive states were selected as representative structures: S1 denotes the structure with slightly separated CO molecules; S2 and S5 are two distinct CO dimerization states; S3 represents two closely approaching CO molecules and S4 presents far separated CO molecules (Fig. 3c). The ML-reproduced atomic positions of CO molecules and the surrounding active sites from S1 to S5 exhibit excellent agreement with the corresponding DFT counterparts. This structural similarity could be quantitatively confirmed with quite low RMSD values (<0.09 Å), implying the tracking of configuration evolution. By combining accurate predictions of spatial structure and energetic properties, our ML protocol presents a cost-effective strategy to monitor dynamic C–C coupling using infrared spectroscopy.

### Tracking the CO–CO dimerization reaction

**Figure 4.**
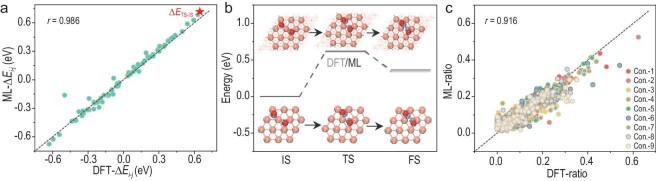
Identification of the energy barriers and the structural information on Cu surface. (a) ML prediction of the energy differences for two random structures from the transition state search of CO–CO dimerization on a pure Cu surface. (b) Comparison of ML-predicted and DFT-computed reaction profiles for CO–CO dimerization. (c) Distribution ratio analysis of nine exemplified configurations by deciphering IR spectroscopy, Con.-1 to Con.-9 stand for the nine configurations obtained from the transition state search.

Concerning the specific CO–CO dimerization reaction involved in C–C coupling evolution, the coupling energy barrier, as well as the configuration variation are two pivotal factors for delineating the reaction characteristics [29,30]. Therefore, these aforementioned features for CO–CO dimerization on Cu surface were investigated with our proposed ML protocol. Configurations generated from transition state search were sampled for IR signals. The ML-predicted energy differences for two random structures among the searching states agree well with those DFT-calculated ones. This alignment signifies accurate monitoring of the energy changes during CO–CO dimerization (Fig. 4a). Meanwhile, the associated configurations are successfully reproduced with high structural consistency (including initial, transition and final states, abbreviated as IS, TS and FS, Fig. S6). Analysis of the energy differences of the corresponding configurations found that the maximum energy difference occurs between the TS and IS (Δ*E*_max_ = Δ*E*_TS-IS_ = *E*_TS_ − *E*_IS_ (IS, initial state; TS, transition state), which coincides with the energy barrier of CO–CO dimerization reaction. Given this, the reaction energy profile, along with the critical atomic positions (CO molecules and surrounding active Cu sites) for IS, TS and FS can be generated via ML predictions, and they show remarkable agreement with DFT-simulated counterparts (Fig. 4b). Specifically, concerning the practical scenarios that multiple structures would concurrently exist during the reaction, leading to spectral mixing rather than singular spectral signals, it is imperative to discern the structure distribution along the reaction pathway. Using the nine configurations derived from the transition state search as the original structures and demonstrations, we systematically investigated the ratios of each configuration by decoding the mixed IR spectroscopy (extra-trees regression ML algorithm, details in Supplementary data). The configuration distribution ratios could be accurately predicted, evidenced by the high Pearson coefficient (*r* = 0.916, Fig. 4c), suggesting the potential to trace the evolution process. With the accurate identification of energy barriers and critical structures, this approach sets a simple and facile strategy to define the reaction profiles in C–C coupling reactions through ML approach.

### Transferability for CO–CO dimerization on Cu-based surfaces

The reaction pathways on unfamiliar surfaces could also be identified with the proposed ML approach. Cu surfaces doped with commonly used transition metals (Fe, Co, Ni, Ag, Au, Ru, Pd, Pt and Ti) and previously reported doping sites (including surface and subsurface positions) were systematically investigated as the perturbations of catalytic active sites for the CO–CO dimerization reaction (Fig. S7) [31–34]. Consequently, various configurations generated from transition state searches on 45 metal surfaces were collected for increasing the ML sample diversity. To improve model transferability and generalization, the transfer learning technique was employed here to leverage knowledge learned from the source task (pre-training task) to solve the target task. Therein, the prior 9 369 datasets collected from the pure Cu system were assigned to pre-training, enabling the model to establish an accurate spectra-structure (input-output) relationship. During the fine-tuning stage, configurations obtained from the transition state searches on 45 metal surfaces were iteratively fed into the model for optimization and calibration, resulting in an enhanced transferability of the model across various systems (see Supplementary data for details).

**Figure 5.**
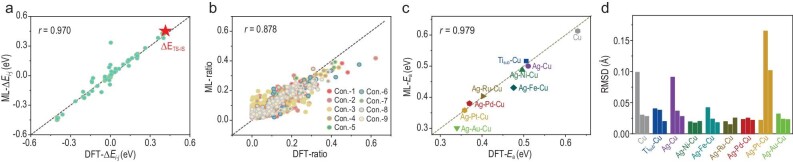
Identification of the energy barriers and the structural information on Cu-based metal surface. (a) ML prediction of the energy differences for two random structures from the transition state search of CO–CO dimerization on Ag-Ru-Cu surface. (b) Configuration distribution ratio analysis by ML deciphering IR spectroscopy. (c) ML prediction performance of energy barriers for validation catalysts. (d) RMSD between the ML-predicted and DFT-simulated configurations for IS, TS and FS of CO–CO dimerization reaction on validation systems.

Within the fine-tuning ML model, the C–C coupling reactions on nine validation catalysts were investigated. As to the more complex Ag-Ru co-doped Cu (Ag-Ru-Cu) surface, the energy barrier and the structural information of critical configurations could be accurately defined (Fig. 5a and Fig. S8). Correspondingly, the reaction energy path for CO–CO dimerization on the Ag-Ru-Cu surface was reproduced, demonstrating the transferability of the trained ML model (Fig. S9). Meanwhile, the configuration distribution ratios show good agreement with the DFT-calculated counterparts, indicating the effectiveness of the ML model in recognizing structure assignment during the reaction (Fig. 5b). Here, we note that Ag doping lowers the energy barrier compared to pure Cu surface, and further co-doping with transition metals such as Ru and Au reduces it even more. This promotion trend for CO–CO dimerization agrees with the literature, confirming the reliability of our ML model (Fig. 5c) [31,35]. Moreover, the RMSD values of IS, TS and FS (final state) on different metal surfaces are generally less than 0.19 Å, demonstrating accurate reproduction of the associated critical structures (Fig. 5d). This suggests that the CO–CO dimerization reaction profile could be elaborately predicted via ML deciphering IR spectroscopy.

## CONCLUSION

We have proposed an ML framework that leverages IR spectroscopy to monitor the evolution behavior of adsorbate-surface interactions. Our approach is exemplified with the C–C coupling process in catalytic reactions, showcasing the ability to accurately trace the dynamic transformations on metal surfaces. When applied this approach to the specific CO–CO dimerization reaction, the critical energy barriers and the corresponding structural information are available. Also, the distribution ratios of the associated configurations could be recognized, allowing the effective tracking of the C–C coupling reaction profile. More broadly, the accessibility of spectroscopic fingerprints through both computational and experimental methods underscores the practicality and versatility of our proposed ML protocol for tracking complex structural evolution. Spectroscopic features serve as a critical bridge linking theoretical predictions with experimental results. Regarding the relative scarcity of experimental spectral data, we initially used cost-effective theoretical simulations to build a basic ML database. By applying the transfer learning technique, our model shows good transferability in predicting the CO–CO dimerization reaction on various Cu-based metallic surfaces. This ML protocol not only enables efficient model training using theoretical spectral data, but also holds promise for further refinement by incorporating experimental data to capture real-world environmental effects. Given the universal applicability of spectroscopic descriptors, combining pre-training with transfer learning technique, this approach offers promising new ways to monitor dynamic chemical processes during chemical evolution via IR spectroscopy.

## METHODS

### *Ab initio* molecular dynamics simulations

AIMD simulations were carried out within the framework of density-functional theory (DFT) as implemented in the CP2K package [36]. The non-empirical Perdew-Burke-Ernzerhof (PBE) generalized gradient approximation was applied to estimate the exchange and correlation potential [37]. To better capture long-range dispersion interactions, we used Grimme's D3 dispersion correction model [38]. The Quickstep method was adopted with an energy cutoff of 500 Ry [39]. We employed the localized double ξ-valence-polarized basis set to expand the wave function [40]. Goedecker-Teter-Hutter pseudopotentials were utilized to model core-electron interactions. We selected the widely researched Cu (111) structure model consisting of 4 layers. On top, solvent water layers were constructed to consider the aqueous environment, as reported in previous studies [31,32]. The temperature for the AIMD simulations was kept constant at 300 K by a Nosé–Hoover thermostat. A cutoff distance of >15 Å along the perpendicular direction was employed to avoid spurious interactions in the *z*-direction. Numerous snapshots were created for structural sampling, using a time step of 0.5 fs. In the source data of Fig. 2, 9 500 configurations were extracted at an even 20 fs interval, and 180 configurations were extracted for sampling infrared spectroscopy in Fig. 3.

### Infrared spectroscopy calculations

As for the spectral calculations, cluster models involving two adsorbed CO molecules and the surrounding active Cu sites were extracted from the entire surface systems, as depicted in Fig. 2a. Hessian calculations were performed using the Gaussian software package [41]. We employed the PBE functional and selected the 6–31 + G* basis set for C and O, and the pseudo LANL2DZ basis set for the metal atoms [42].

### Transition state search

As to the configurations for CO–CO dimerization reaction, we conducted various transition state search calculations via the CP2K package [36]. The structural models included the pristine Cu surface and the doped structures. The dopant atom positions (Fe, Co, Ni, Ag, Au, Ru, Pd, Pt and Ti) were constructed according to the previous literatures [31,32]. Water layers were considered in the transition state searches of CO–CO dimerization to account for solvation effects [24]. The transition states were identified by using the climbing image-nudged elastic band method [43].

## Supplementary Material

nwae389_Supplemental_File

## Data Availability

The code for the machine learning model is provided at https://doi.org/10.5281/zenodo.8382444. The source data in this article can be found at https://doi.org/10.5281/zenodo.8415393.
