# Supplementary material for: Monitoring C–C coupling in catalytic reactions via machine-learned infrared spectroscopy
Source: Natl Sci Rev. 2024 Nov 4;12(2):nwae389. doi: 10.1093/nsr/nwae389 (PMC11737394; doi:10.1093/nsr/nwae389)
Supplement: nwae389_Supplemental_File [file nwae389_supplemental_file.pdf]

Supplementary data for

Monitoring C-C coupling in catalytic reactions via machine-learned infrared spectroscopy

Li Yang[1,2,3,†], Zhicheng Zhao[1,†], Tongtong Yang[4,†], Donglai Zhou[4], Xiaoyu Yue[4], Xiyu Li[4], Yan Huang[4], Xijun Wang[4], Ruyun Zheng[1], Thomas Heine[2,3,]*, Changyin Sun[1,]*, Jun Jiang[4,]* and Sheng Ye[1,]*

1. Institutes of Physical Science and Information Technology, Engineering Research Center of Autonomous Unmanned System Technology, Ministry of Education, Anhui Provincial Engineering Research Center for Unmanned System and Intelligent Technology, School of Artificial Intelligence, Anhui University, Hefei, Anhui 230601, China.

2. Helmholtz-Zentrum Dresden-Rossendorf, Bautzner Landstrasse 400, Dresden 01328, Germany.

3. Theoretical Chemistry, Technische Universität Dresden, Mommsenstr. 13, Dresden 01062, Germany.

4. Key Laboratory of Precision and Intelligent Chemistry, School of Chemistry and Materials Science, University of Science and Technology of China, Hefei, Anhui, 230088, China.

*Corresponding authors emails: thomas.heine@tu-dresden.de; cysun@ahu.edu.cn; jiangj1@ustc.edu.cn; yess@mail.ustc.edu.cn

† Equally contributed to this work.

Machine learning protocol

In the machine learning (ML) process, the dataset was partitioned into a 9:1 ratio for training and testing purposes. A convolutional neural network (CNN) model was implanted in our task, which consists of multiple CNN blocks and a fully connected layer. Each CNN block contains three branches, with a convolutional layer, a ReLU activation function, a max pooling layer, and a batch normalization layer [1]. The convolutional kernels in the three branches have sizes of 5, 7, and 9, respectively, with a stride of 1 and padding sizes of 0, 1, and 2.

The three branches of each CNN block were concatenated along the channel dimension, and a 1x1 convolutional layer was applied to reduce the number of output channels. A squeeze-and-excitation (SE) block was employed to the output of the CNN blocks, involving a global average pooling layer and followed by two fully connected layers together with a sigmoid activation function [2]. The SE block was used to selectively weight the importance of each feature map.

PyTorch deep learning framework was adopted to implement our model, and we used the Adam optimizer with a learning rate of 0.001 and a batch size of 128 to train this model [3]. L1 loss was used as the objective function for the regression task. The model was trained for 300 epochs, and the best model was selected based on the validation loss.

During the training process, to alleviate domain discrepancies, the MixUp technique was employed. This approach is a data augmentation strategy that generates new training samples by interpolating between two randomly chosen samples and their corresponding labels [4]. It could encourage the model to exhibit smoother behavior of different domains, reducing the impact of domain differences and enhancing the generalization capabilities [5].

Considering the coexistence of multiple structures during C-C coupling process, the configuration distribution ratios were predicted by deciphering the mixed spectra. Extra-trees regression algorithm implemented in Scikit-learn framework composed of 300 decision trees was used [6], in which, 5,000 mixed spectra were generated to be the machine learning dataset.

The accuracy and robustness of the machine learning predictions were evaluated using a cross-validation technique. The dataset was randomly divided into ten equal-sized subsets. In each iteration, one subset was used as the test set, while the remaining nine subsets were used for training. This process was repeated for all subsets to ensure the reliable performance assessment.

For transfer learning, various configurations generated from transition state searches on 45 Cu and Cu-based metal surfaces were collected. During the fine-tuning step, pre-trained model weights were loaded to optimize the prediction for transferred metal surfaces. The optimizer and learning rate strategy remained consistent with the pre-training step, and weights were updated across all layers.

Subsequently, the dataset comprising 36 metal surfaces (108 configurations) was utilized to construct the fine-tuning dataset, which was randomly split into training and test sets in a ratio of 8:2. The remaining 9 metal surfaces (27 configurations) served as a validation set to evaluate the performance of the fine-tuned model.

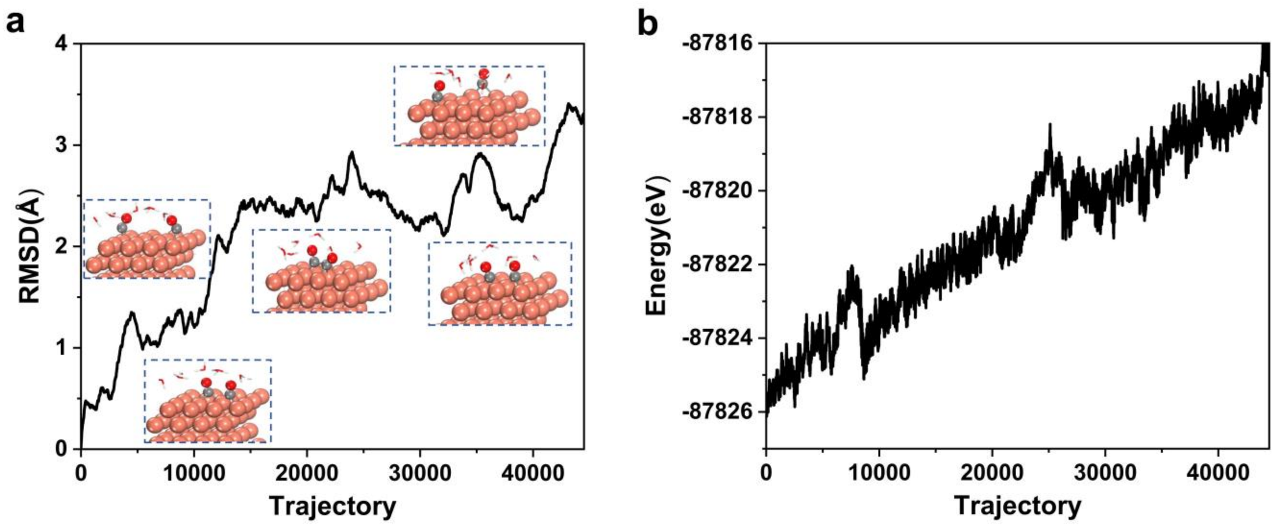

Fig. S1. Structural and energetic discrepancy of the ab initio molecular dynamics (AIMD) trajectories. (a) The Root mean square deviation (RMSD) and energy deviations (b) of the 45,000 trajectories obtained from AIMD simulations. As to the RMSD calculation, the first trajectory was used as the reference configuration.

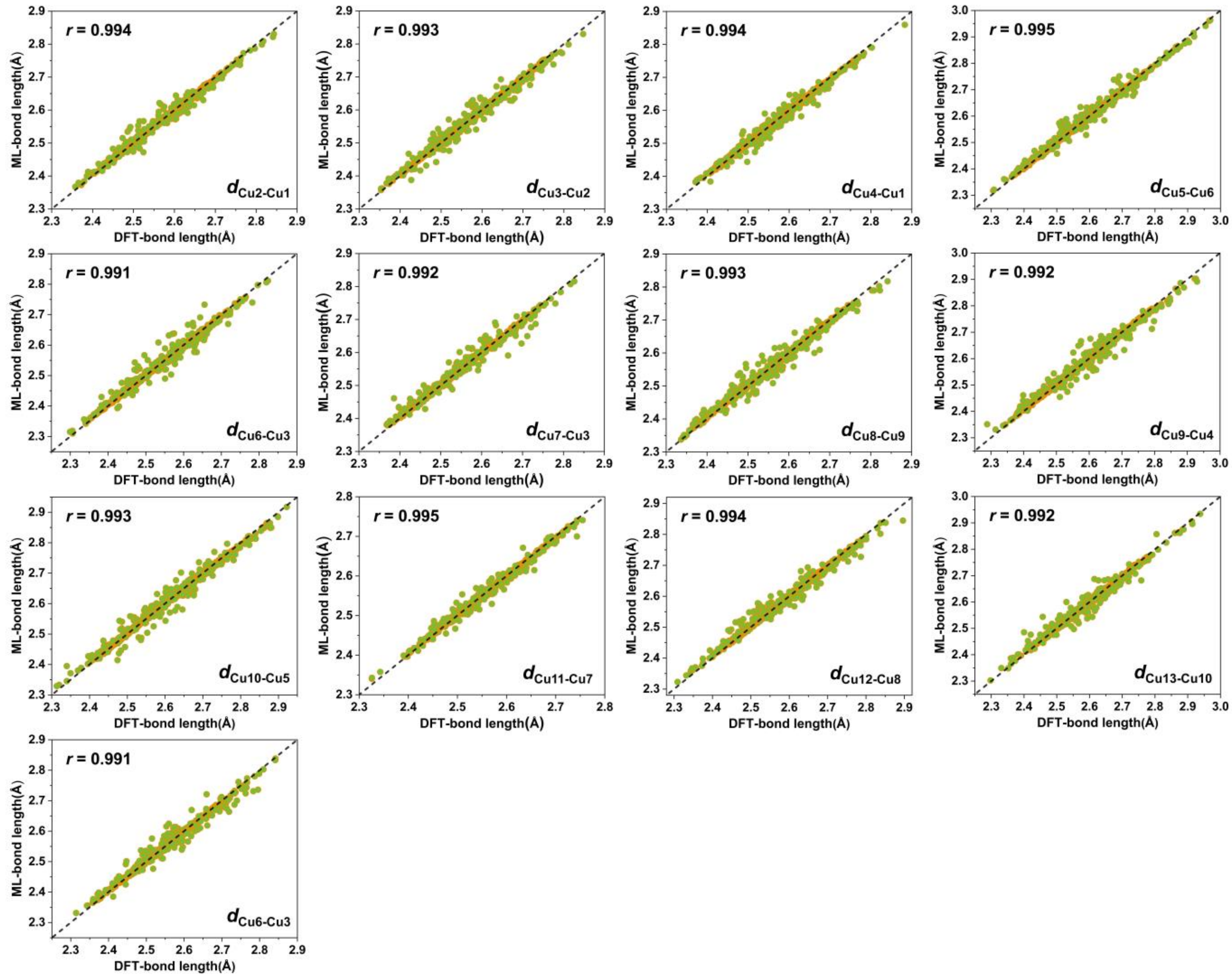

Fig. S2. ML prediction of the most relevant bond length information for the surrounding Cu active sites.

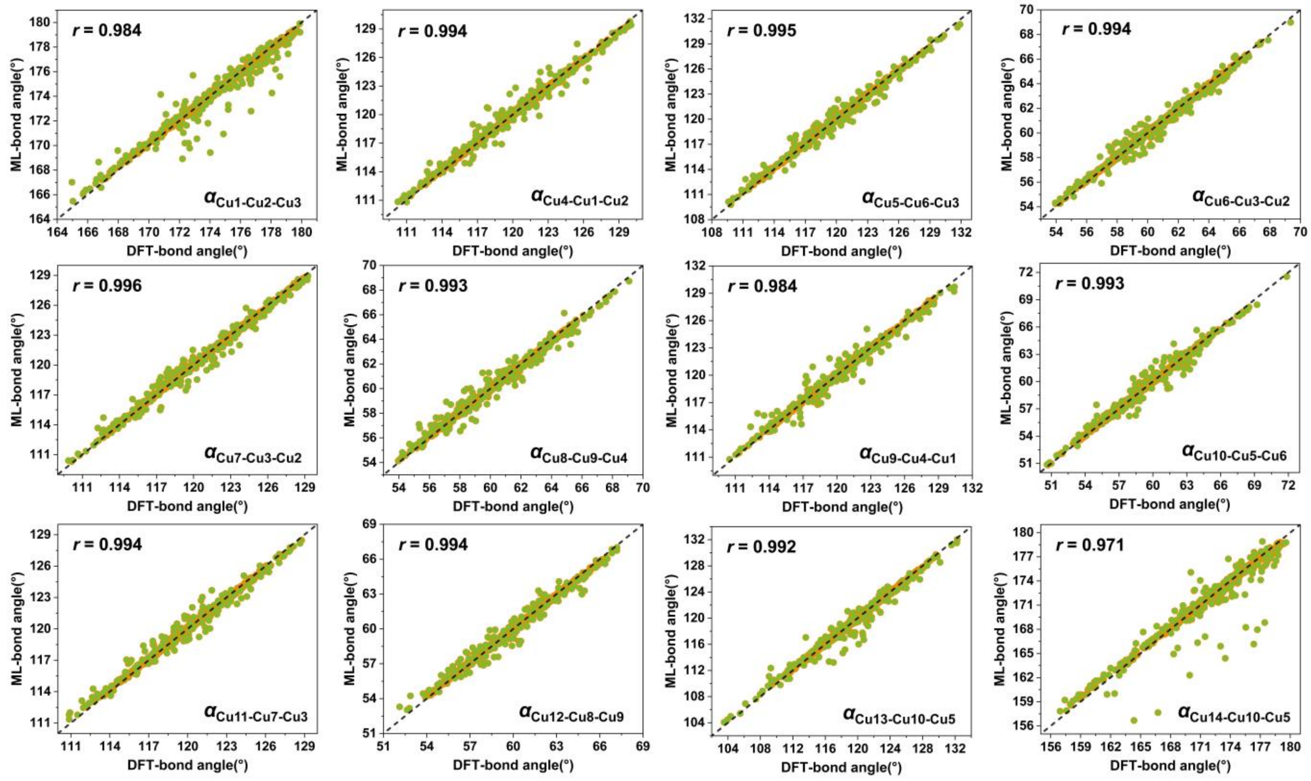

Fig. S3. ML prediction of the most relevant bond angle information for the surrounding Cu active sites.

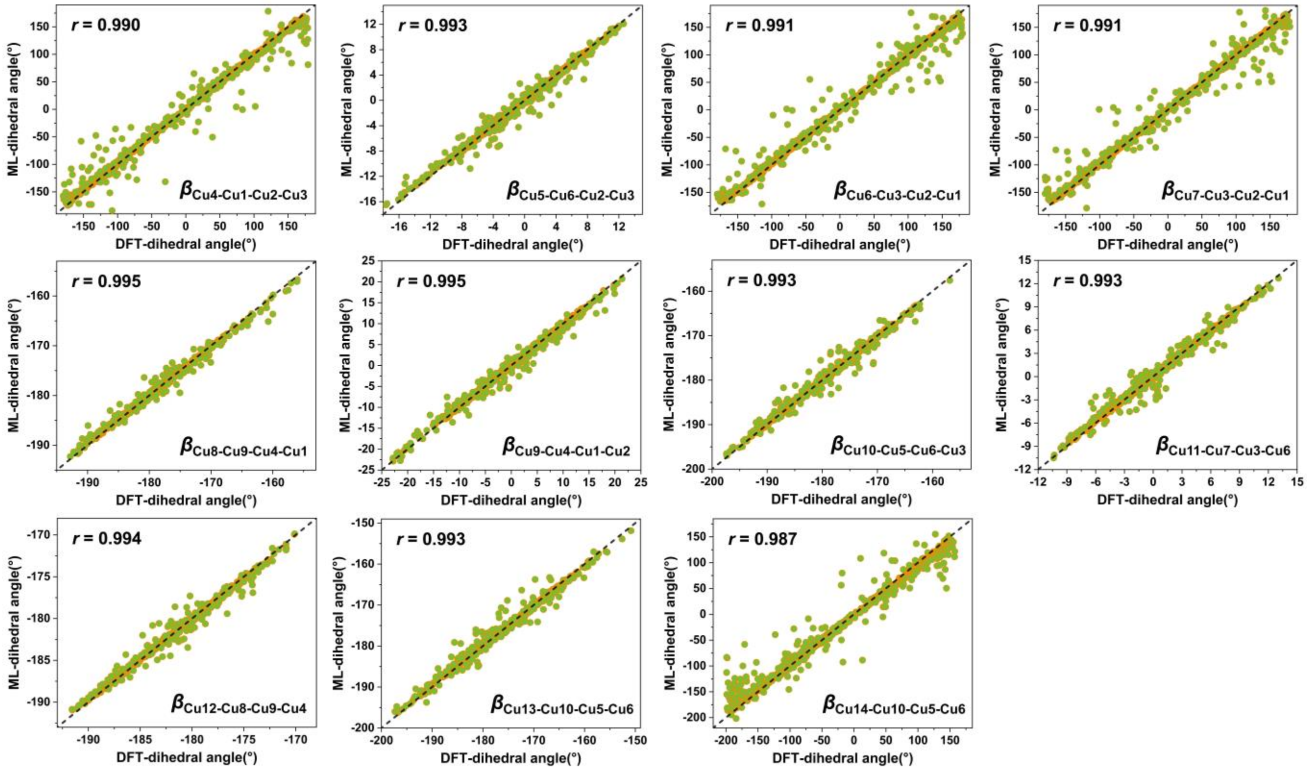

Fig. S4. ML prediction of the most relevant dihedral angle information for the surrounding Cu active sites.

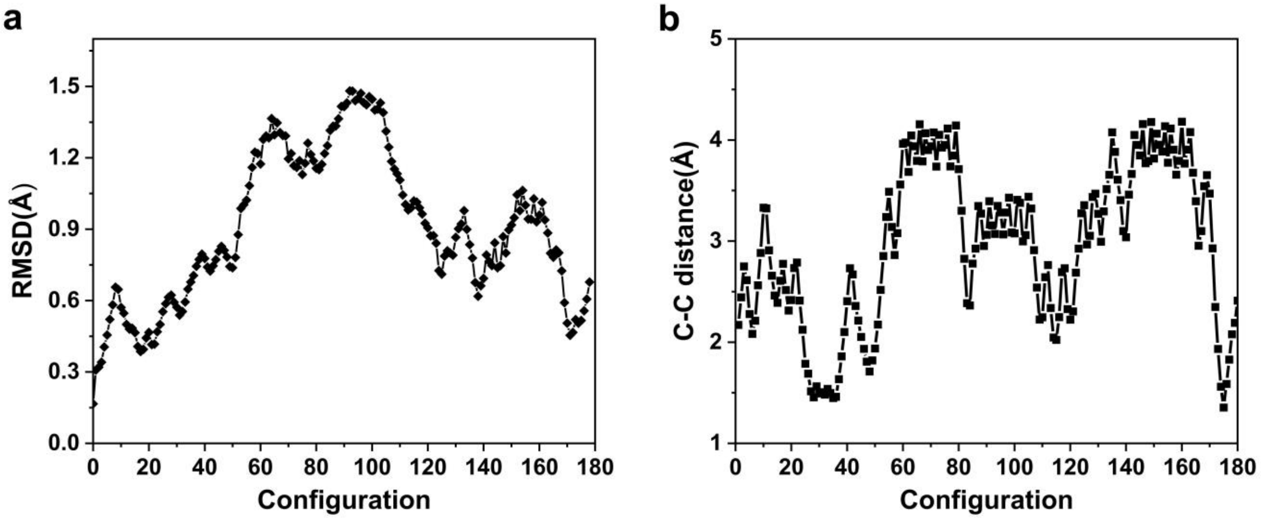

Fig. S5. Configuration deviation of CO molecules. (a) Root mean square deviation (RMSD) of two adsorbed CO molecules for the extracted 180 configurations, the first configuration was selected as the reference structure. (b) The distance variations between two carbon atoms of the adsorbed CO molecules.

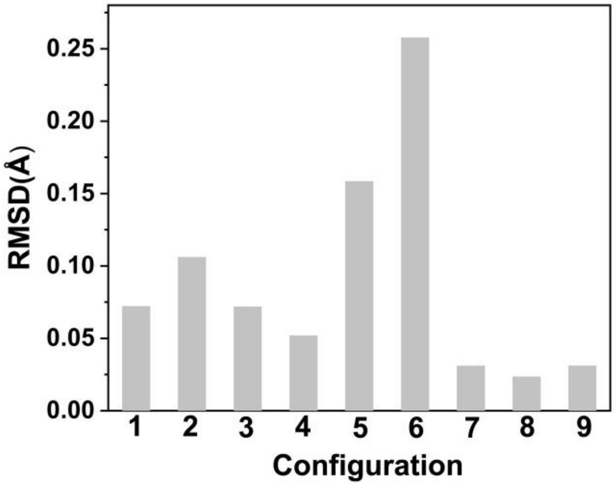

Fig. S6. Structural discrepancy of ML predicted and DFT simulated configurations on Cu surface. The root mean square deviation (RMSD) between ML-predicted configurations and the DFT-simulated ones for the nine structures obtained from the transition state search on Cu metal surface.

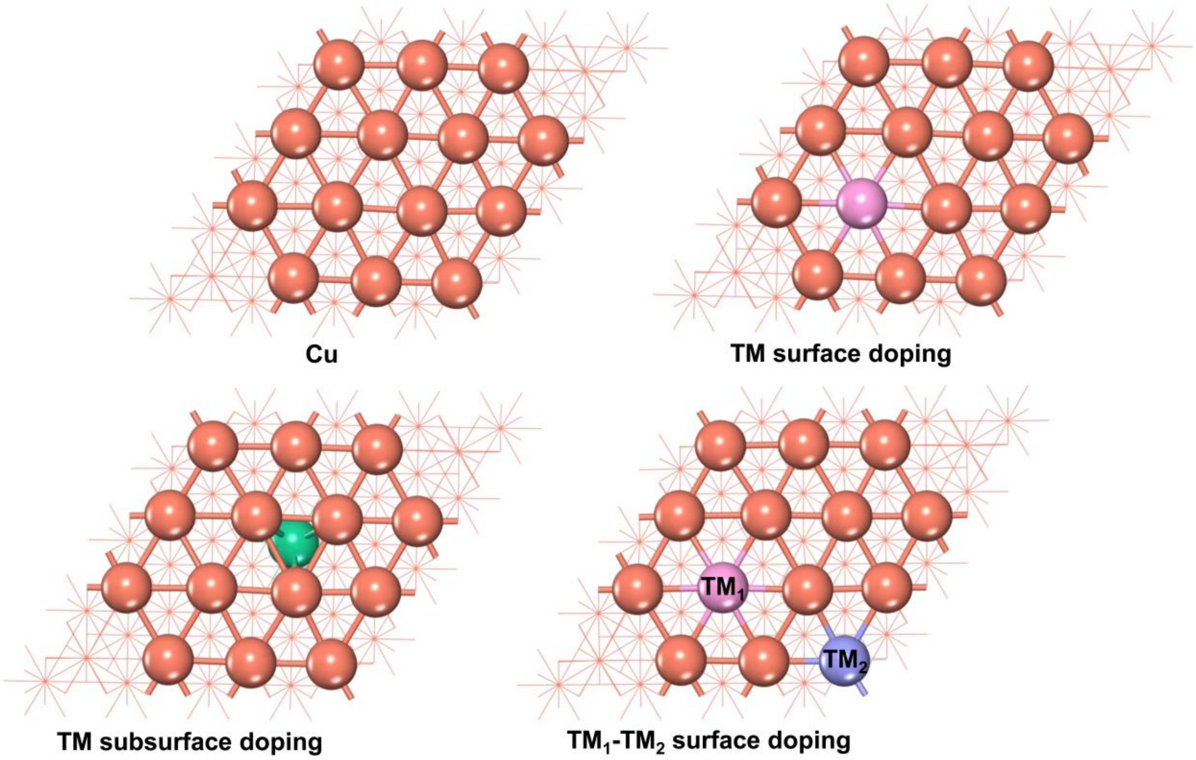

Fig. S7. Atomic representations of the studied Cu and Cu-based metal surfaces. Configurations for the pristine Cu surface, surface doping of Cu with transition metals (Fe, Co, Ni, Ag, Au, Ru, Pd, Pt), subsurface doping of Cu (Fe, Co, Ni, Ag, Au, Ru, Pd, Pt and Ti), and dual metal doping of Cu systems. TM denotes transition metal, TM_1 and TM_2 are one metal atom among Fe, Co, Ni, Ag, Au, Ru, Pd, Pt. The doping atom positions and metals were arranged according to the previous literatures [7,8].

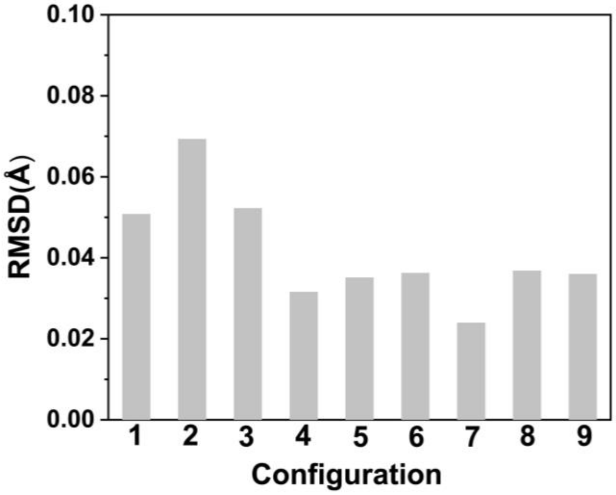

Fig. S8. Structural discrepancy of ML predicted and DFT simulated configurations on Ag-Ru-Cu surface. The root mean square deviation (RMSD) between the ML-predicted configurations and the DFT-simulated ones for the nine structures obtained from the transition state search on Ag-Ru-Cu metal surface.

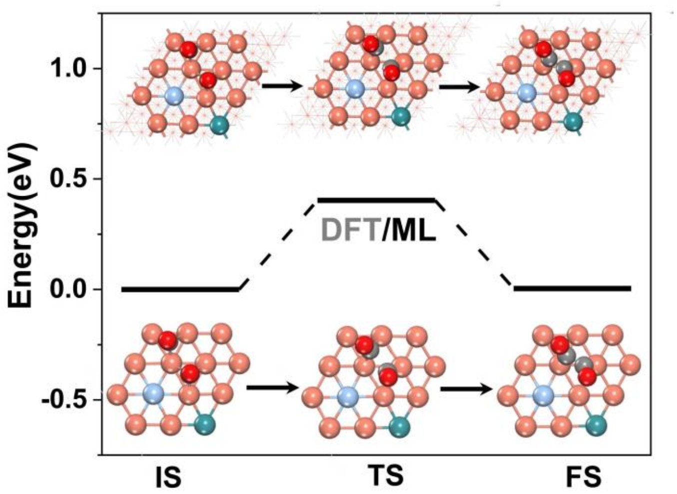

Fig. S9. CO-CO dimerization reaction profile on Ag-Ru-Cu-surface. Comparison of ML-predicted and DFT-computed reaction profiles for CO-CO dimerization on Ag-Ru-Cu surface.

REFERENCES

1. Szegedy C, Vanhoucke V, Ioffe S et al. Rethinking the inception architecture for computer vision. In Proceedings of the IEEE Conference on Computer Vision and Pattern Recognition 2016; 2818–26.
2. Hu J, Shen L, Sun G et al. Squeeze-and-excitation networks. In Proceedings of the IEEE/CVF Conference on Computer Vision and Pattern Recognition 2018; 7132-41.
3. Kingma D and Ba J. Adam: A method for stochastic optimization. Presented at the 3rd International Conference on Learning Representations, San Diego, CA, 2015.
4. Yao H, Wang Y, Zhang L et al. C-Mixup: Improving generalization in regression. arXiv 2022.
5. Zhou Q, Feng Z, Gu Q et al. Context-aware mixup for domain adaptive semantic segmentation. IEEE Trans Circuits Syst Video Technol 2023; **33:** 804-17.
6. Fabian P, Pedregosa F, Varoquaux G et al. Scikit-learn: Machine learning in Python. J Mach Learn Res 2011; **12:** 2825-30.
7. Wang X, Ou P, Ozden A et al. Efficient electrosynthesis of n-propanol from carbon monoxide using a Ag-Ru-Cu catalyst.. *Nat Energy* 2022; **7:** 170-6.
8. Hu F, Yang L, Jiang Y et al. Ultrastable Cu catalyst for CO_2 electroreduction to multicarbon liquid fuels by tuning C-C coupling with CuTi subsurface. *Angew Chem Int Ed* 2021; **133:** 26326-31.
